# Supplementary material for: Effect of Dancing Interventions on Depression and Anxiety Symptoms in Older Adults: A Systematic Review and Meta-Analysis
Source: Behav Sci (Basel). 2024 Jan 10;14(1):43. doi: 10.3390/bs14010043 (PMC10813489; doi:10.3390/bs14010043)
Supplement: Supplementary file 1 [file behavsci-14-00043-s001.zip › behavsci-2744560-supplementary.pdf]

Table S1. Comprehensive search strategy customized for each database and corresponding number of search results retrieved.

Database	Search strategy	Results October 12th 2023
**Medline / PubMed**	("Dancing"[MeSH Terms] OR "Dancing"[All Fields] OR "Dance"[All Fields] OR "danced"[All Fields] OR "dances"[All Fields] OR "dancers"[All Fields] OR "Ballet"[All Fields] OR "Square Dance"[All Fields] OR "hip hop"[All Fields] OR "Jazz"[All Fields] OR "Tap Dance"[All Fields] OR "Modern Dance"[All Fields] OR "Salsa"[All Fields] OR "Line Dancing"[All Fields] OR "social dance"[All Fields] OR "Tango"[All Fields] OR "ballroom"[All Fields] OR "Irish set"[All Fields] OR "Square Dance"[All Fields] OR "waltz"[All Fields] OR "Aerobic dancing"[All Fields] OR "Ballroom dancing"[All Fields] OR "Belly dance"[All Fields] OR "Break dancing"[All Fields] OR "Cha-cha"[All Fields] OR "Flamenco"[All Fields] OR "Folk dancing"[All Fields] OR "Ice dancing"[All Fields] OR "Pole dancing"[All Fields] OR "Step dancing"[All Fields] OR "Rumba"[All Fields] OR "Blues"[All Fields] OR "Jitterbug"[All Fields] OR "Viennese"[All Fields] OR "Foxtrot"[All Fields] OR "Swing"[All Fields] OR "Merengue"[All Fields] OR "Disco"[All Fields] OR "Dance Therapy"[MeSH Terms] OR "Dance Therapy"[All Fields] OR "Dance Therapies"[All Fields] OR "therapeutic dance"[All Fields] OR "Movement Therapy"[All Fields] OR "Dance movement therapy"[All Fields]) AND ("Depression"[MeSH Terms] OR "Depression"[Title/Abstract] OR "Depressive Symptoms"[All Fields] OR "Depressive Symptom"[All Fields] OR "Emotional Depression"[All Fields] OR "central depression"[All Fields] OR "clinical depression"[All Fields] OR "depressive disease"[All Fields] OR "Depressive Disorder"[All Fields] OR "depressive episode"[All Fields] OR "depressive illness"[All Fields] OR "depressive personality disorder"[All Fields] OR "depressive state"[All Fields] OR "Depressive Symptom"[All Fields] OR "Depressive Syndrome"[All Fields] OR "mental depression"[All Fields] OR "parental depression"[All Fields] OR "Major Depression"[All Fields] OR "late life depression"[All Fields] OR "late life depression"[All Fields] OR "Depressive Disorder"[MeSH Terms] OR "Depressive Disorder"[All Fields] OR "Depressive Disorders"[All Fields] OR "Depressive Neuroses"[All Fields] OR "Depressive Neurosis"[All Fields] OR "Endogenous Depression"[All Fields] OR "Endogenous Depressions"[All Fields] OR "Depressive Syndrome"[All Fields] OR "Depressive Syndromes"[All Fields] OR "Neurotic Depression"[All Fields] OR "Neurotic Depressions"[All Fields] OR "Melancholia"[All Fields] OR "Melancholias"[All Fields] OR "Unipolar Depression"[All Fields] OR "Unipolar Depressions"[All Fields] OR "depressive disorder, major"[MeSH Terms] OR "Major Depressive Disorders"[All Fields] OR "Major Depressive Disorder"[All Fields] OR "Involutional Psychoses"[All Fields] OR "Involutional Psychosis"[All Fields] OR "Involutional Depression"[All Fields] OR "Involutional Melancholia"[All Fields] OR "Anxiety"[MeSH Terms] OR "Anxiety"[Title/Abstract] OR "Angst"[All Fields] OR "Social Anxiety"[All Fields] OR "Social Anxieties"[All Fields] OR "Hypervigilance"[All Fields] OR "Nervousness"[All Fields] OR "Anxiousness"[All Fields] OR "Apprehension"[All Fields] OR "Worry"[All Fields] OR "Anxiety Disorders"[MeSH Terms] OR "Anxiety Disorders"[All Fields] OR "Anxiety Disorder"[All Fields] OR "Anxiety Neuroses"[All Fields] OR "Neurotic Anxiety States"[All Fields] OR "Anxiety Neurosis"[All Fields]) AND ("Aged"[MeSH Terms] OR "Aged"[All Fields] OR "Elderly"[All Fields] OR "aged, 80 and over"[MeSH Terms] OR "80 and over"[All Fields] OR "Oldest Old"[All Fields] OR "Nonagenarian"[All Fields] OR "Nonagenarians"[All Fields] OR "Octogenarians"[All Fields] OR "Octogenarian"[All Fields] OR "Centenarians"[All Fields] OR "Centenarian"[All Fields] OR "geriatric"[Title/Abstract] OR "Middle Aged"[MeSH Terms] OR "Middle Aged"[All Fields] OR "Middle Age"[All Fields])	526
**Embase**	('dancing'/de OR dancing OR 'dance'/de OR dance OR danced OR dances OR dancers OR 'ballet'/de OR ballet OR 'hip hop' OR 'jazz'/de OR jazz OR 'tap dance' OR 'modern dance' OR 'salsa'/de OR salsa OR 'line dancing' OR 'social dance' OR 'tango'/de OR tango OR ballroom OR 'irish set' OR 'square dance' OR waltz OR 'aerobic dancing'/de OR 'aerobic dancing' OR 'ballroom dancing' OR 'belly dance' OR 'break dancing'/de OR 'break dancing' OR 'cha-cha' OR flamenco OR 'folk dancing' OR 'ice dancing' OR 'pole dancing' OR 'step dancing' OR rumba OR blues OR jitterbug OR viennese OR foxtrot OR 'swing'/de OR swing OR merengue OR disco OR 'dance therapy'/de OR 'dance therapy' OR 'dance therapies' OR 'therapeutic dance' OR 'movement therapy'/de OR 'movement therapy' OR 'dance movement therapy'/de OR 'dance movement therapy') AND ('depression'/de OR depression OR 'depressive symptoms'/de OR 'depressive symptoms' OR 'emotional depression' OR 'central depression'/de OR 'central depression' OR 'clinical depression'/de OR 'clinical depression' OR 'depressive disease'/de OR 'depressive disease' OR 'depressive episode'/de OR 'depressive episode' OR 'depressive illness'/de OR 'depressive illness' OR 'depressive personality disorder'/de OR 'depressive personality disorder' OR 'depressive state'/de OR 'depressive state' OR 'depressive symptom'/de OR 'depressive symptom'	1,448

	OR 'mental depression'/de OR 'mental depression' OR 'parental depression'/de OR 'parental depression' OR 'major depression'/de OR 'major depression' OR 'late life depression'/de OR 'late life depression' OR 'depressive disorder'/de OR 'depressive disorder' OR 'depressive disorders' OR 'depressive neuroses' OR 'depressive neurosis'/de OR 'depressive neurosis' OR 'endogenous depression'/de OR 'endogenous depression' OR 'endogenous depressions' OR 'depressive syndrome'/de OR 'depressive syndrome' OR 'depressive syndromes' OR 'neurotic depression'/de OR 'neurotic depression' OR 'neurotic depressions' OR 'melancholia'/de OR 'melancholia' OR 'melancholias' OR 'unipolar depression'/de OR 'unipolar depression' OR 'unipolar depressions' OR 'major depressive disorders' OR 'major depressive disorder'/de OR 'major depressive disorder' OR 'involutional psychoses' OR 'involutional psychosis'/de OR 'involutional psychosis' OR 'involutional depression'/de OR 'involutional depression' OR 'involutional melancholia'/de OR 'involutional melancholia' OR 'anxiety'/de OR anxiety OR angst OR 'social anxiety'/de OR 'social anxiety' OR 'social anxieties' OR 'hypervigilance'/de OR hypervigilance OR 'nervousness'/de OR nervousness OR anxiousness OR 'apprehension'/de OR apprehension OR 'worry'/de OR worry OR 'anxiety disorders'/de OR 'anxiety disorders' OR 'anxiety disorder'/de OR 'anxiety disorder' OR 'anxiety neuroses' OR 'neurotic anxiety states' OR 'anxiety neurosis'/de OR 'anxiety neurosis' OR 'neurotic anxiety state' OR 'involutional paraphrenia' OR 'involutional paraphrenias') AND ('aged'/de OR aged OR 'elderly'/de OR elderly OR '80 and over' OR 'oldest old' OR 'nonagenarian'/de OR nonagenarian OR 'nonagenarians'/de OR nonagenarians OR 'octogenarians'/de OR octogenarians OR 'octogenarian'/de OR octogenarian OR 'centenarians'/de OR centenarians OR 'centenarian'/de OR centenarian OR 'geriatric'/de OR geriatric OR 'middle aged'/de OR 'middle aged' OR 'middle age'/de OR 'middle age')	
**Scopus**	TITLE-ABS-KEY(Dancing OR Dance OR danced OR dances OR dancers OR Ballet OR "Square Dance" OR "hip hop" OR Jazz OR "Tap Dance" OR "Modern Dance" OR Salsa OR "Line Dancing" OR "social dance" OR Tango OR ballroom OR "Irish set" OR "Square Dance" OR waltz OR "Aerobic dancing" OR "Ballroom dancing" OR "Belly dance" OR "Break dancing" OR "Cha-cha" OR Flamenco OR "Folk dancing" OR "Ice dancing" OR "Pole dancing" OR "Step dancing" OR Rumba OR Blues OR Jitterbug OR Viennese OR Foxtrot OR Swing OR Merengue OR Disco OR "Dance Therapy" OR "Dance Therapies" OR "therapeutic dance" OR "Movement Therapy" OR "Dance movement therapy") AND TITLE-ABS-KEY(Depression OR "Depressive Symptoms" OR "Depressive Symptom" OR "Emotional Depression" OR "central depression" OR "clinical depression" OR "depressive disease" OR "Depressive Disorder" OR "depressive episode" OR "depressive illness" OR "depressive personality disorder" OR "depressive state" OR "Depressive Symptom" OR "Depressive Syndrome" OR "mental depression" OR "parental depression" OR "Major Depression" OR "late life depression" OR "late life depression" OR "Depressive Disorder" OR "Depressive Disorders" OR "Depressive Neuroses" OR "Depressive Neurosis" OR "Endogenous Depression" OR "Endogenous Depressions" OR "Depressive Syndrome" OR "Depressive Syndromes" OR "Neurotic Depression" OR "Neurotic Depressions" OR "Melancholia" OR "Melancholias" OR "Unipolar Depression" OR "Unipolar Depressions" OR "Major Depressive Disorders" OR "Major Depressive Disorder" OR "Involutional Psychoses" OR "Involutional Psychosis" OR "Involutional Depression" OR "Involutional Melancholia" OR Anxiety OR Angst OR "Social Anxiety" OR "Social Anxieties" OR Hypervigilance OR Nervousness OR Anxiousness OR Apprehension OR Worry OR "Anxiety Disorders" OR "Anxiety Disorder" OR "Anxiety Neuroses" OR "Neurotic Anxiety States" OR "Anxiety Neurosis" OR "Neurotic Anxiety State" OR "Involutional Paraphrenia" OR "Involutional Paraphrenias") AND TITLE-ABS-KEY(Aged OR Elderly OR "80 and over" OR "Oldest Old" OR Nonagenarian OR Nonagenarians OR Octogenarians OR Octogenarian OR Centenarians OR Centenarian OR geriatric OR "Middle Aged" OR "Middle Age")	811
**Web of Science**	TS=(Dancing OR Dance OR danced OR dances OR dancers OR Ballet OR "Square Dance" OR "hip hop" OR Jazz OR "Tap Dance" OR "Modern Dance" OR Salsa OR "Line Dancing" OR "social dance" OR Tango OR ballroom OR "Irish set" OR "Square Dance" OR waltz OR "Aerobic dancing" OR "Ballroom dancing" OR "Belly dance" OR "Break dancing" OR "Cha-cha" OR Flamenco OR "Folk dancing" OR "Ice dancing" OR "Pole dancing" OR "Step dancing" OR Rumba OR Blues OR Jitterbug OR Viennese OR Foxtrot OR Swing OR Merengue OR Disco OR "Dance Therapy" OR "Dance Therapies" OR "therapeutic dance" OR "Movement Therapy" OR "Dance movement therapy") AND TS=(Depression OR "Depressive Symptoms" OR "Depressive Symptom" OR "Emotional Depression" OR "central depression" OR "clinical depression" OR "depressive disease" OR "Depressive Disorder" OR "depressive episode" OR "depressive illness" OR "depressive personality disorder" OR "depressive state" OR "Depressive Symptom" OR "Depressive Syndrome" OR "mental depression" OR "parental depression" OR "Major Depression" OR "late life depression" OR "late life depression" OR "Depressive Disorder" OR "Depressive Disorders" OR	893

	"Depressive Neuroses" OR "Depressive Neurosis" OR "Endogenous Depression" OR "Endogenous Depressions" OR "Depressive Syndrome" OR "Depressive Syndromes" OR "Neurotic Depression" OR "Neurotic Depressions" OR "Melancholia" OR "Melancholias" OR "Unipolar Depression" OR "Unipolar Depressions" OR "Major Depressive Disorders" OR "Major Depressive Disorder" OR "Involutional Psychoses" OR "Involutional Psychosis" OR "Involutional Depression" OR "Involutional Melancholia" OR Anxiety OR Angst OR "Social Anxiety" OR "Social Anxieties" OR Hypervigilance OR Nervousness OR Anxiousness OR Apprehension OR Worry OR "Anxiety Disorders" OR "Anxiety Disorder" OR "Anxiety Neuroses" OR "Neurotic Anxiety States" OR "Anxiety Neurosis" OR "Neurotic Anxiety State" OR "Involutional Paraphrenia" OR "Involutional Paraphrenias") AND TS=(Aged OR Elderly OR "80 and over" OR "Oldest Old" OR Nonagenarian OR Nonagenarians OR Octogenarians OR Octogenarian OR Centenarians OR Centenarian OR geriatric OR "Middle Aged" OR "Middle Age")	
**PsycINFO**	((Keywords: (Dancing) OR Keywords: (Dance) OR Keywords: (danced) OR Keywords: (dances) OR Keywords: (dancers) OR Keywords: (Ballet) OR Keywords: ("Square Dance") OR Keywords: ("hip hop") OR Keywords: (Jazz) OR Keywords: ("Tap Dance") OR Keywords: ("Modern Dance") OR Keywords: (Salsa) OR Keywords: ("Line Dancing") OR Keywords: ("social dance") OR Keywords: (Tango) OR Keywords: (ballroom) OR Keywords: ("Irish set") OR Keywords: ("Square Dance") OR Keywords: (waltz) OR Keywords: ("Aerobic dancing") OR Keywords: ("Ballroom dancing") OR Keywords: ("Belly dance") OR Keywords: ("Break dancing") OR Keywords: ("Cha-cha") OR Keywords: (Flamenco) OR Keywords: ("Folk dancing") OR Keywords: ("Ice dancing") OR Keywords: ("Pole dancing") OR Keywords: ("Step dancing") OR Keywords: (Rumba) OR Keywords: (Blues) OR Keywords: (Jitterbug) OR Keywords: (Viennese) OR Keywords: (Foxtrot) OR Keywords: (Swing) OR Keywords: (Merengue) OR Keywords: (Disco) OR Keywords: ("Dance Therapy") OR Keywords: ("Dance Therapies") OR Keywords: ("therapeutic dance") OR Keywords: ("Movement Therapy") OR Keywords: ("Dance movement therapy")) AND (Keywords: (Depression) OR Keywords: ("Depressive Symptoms") OR Keywords: ("Depressive Symptom") OR Keywords: ("Emotional Depression") OR Keywords: ("central depression") OR Keywords: ("clinical depression") OR Keywords: ("depressive disease") OR Keywords: ("Depressive Disorder") OR Keywords: ("depressive episode") OR Keywords: ("depressive illness") OR Keywords: ("depressive personality disorder") OR Keywords: ("depressive state") OR Keywords: ("Depressive Symptom") OR Keywords: ("Depressive Syndrome") OR Keywords: ("mental depression") OR Keywords: ("parental depression") OR Keywords: ("Major Depression") OR Keywords: ("late life depression") OR Keywords: ("late life depression") OR Keywords: ("Depressive Disorder") OR Keywords: ("Depressive Disorders") OR Keywords: ("Depressive Neuroses") OR Keywords: ("Depressive Neurosis") OR Keywords: ("Endogenous Depression") OR Keywords: ("Endogenous Depressions") OR Keywords: ("Depressive Syndrome") OR Keywords: ("Depressive Syndromes") OR Keywords: ("Neurotic Depression") OR Keywords: ("Neurotic Depressions") OR Keywords: ("Melancholia") OR Keywords: ("Melancholias") OR Keywords: ("Unipolar Depression") OR Keywords: ("Unipolar Depressions") OR Keywords: ("Major Depressive Disorders") OR Keywords: ("Major Depressive Disorder") OR Keywords: ("Involutional Psychoses") OR Keywords: ("Involutional Psychosis") OR Keywords: ("Involutional Depression") OR Keywords: ("Involutional Melancholia") OR Keywords: (Anxiety) OR Keywords: (Angst) OR Keywords: ("Social Anxiety") OR Keywords: ("Social Anxieties") OR Keywords: (Hypervigilance) OR Keywords: (Nervousness) OR Keywords: (Anxiousness) OR Keywords: (Apprehension) OR Keywords: (Worry) OR Keywords: ("Anxiety Disorders") OR Keywords: ("Anxiety Disorder") OR Keywords: ("Anxiety Neuroses") OR Keywords: ("Neurotic Anxiety States") OR Keywords: ("Anxiety Neurosis") OR Keywords: ("Neurotic Anxiety State") OR Keywords: ("Involutional Paraphrenia") OR Keywords: ("Involutional Paraphrenias")) AND (Keywords: (Aged) OR Keywords: (Elderly) OR Keywords: ("80 and over") OR Keywords: ("Oldest Old") OR Keywords: (Nonagenarian) OR Keywords: (Nonagenarians) OR Keywords: (Octogenarians) OR Keywords: (Octogenarian) OR Keywords: (Centenarians) OR Keywords: (Centenarian) OR Keywords: (geriatric) OR Keywords: ("Middle Aged") OR Keywords: ("Middle Age"))) OR ((abstract: (Dancing) OR abstract: (Dance) OR abstract: (danced) OR abstract: (dances) OR abstract: (dancers) OR abstract: (Ballet) OR abstract: ("Square Dance") OR abstract: ("hip hop") OR abstract: (Jazz) OR abstract: ("Tap Dance") OR abstract: ("Modern Dance") OR abstract: (Salsa) OR abstract: ("Line Dancing") OR abstract: ("social dance") OR abstract: (Tango) OR abstract: (ballroom) OR abstract: ("Irish set") OR abstract: ("Square Dance") OR abstract: (waltz) OR abstract: ("Aerobic dancing") OR abstract: ("Ballroom dancing") OR abstract: ("Belly dance") OR abstract: ("Break dancing") OR abstract: ("Cha-cha") OR abstract: (Flamenco) OR abstract: ("Folk dancing") OR abstract: ("Ice dancing") OR abstract: ("Pole dancing") OR abstract: ("Step dancing") OR abstract: (Rumba) OR abstract: (Blues) OR abstract: (Jitterbug) OR abstract: (Viennese) OR abstract: (Foxtrot) OR abstract: (Swing) OR abstract: (Merengue) OR abstract: (Disco) OR abstract: ("Dance	95

	Therapy") OR abstract: ("Dance Therapies") OR abstract: ("therapeutic dance") OR abstract: ("Movement Therapy") OR abstract: ("Dance movement therapy")) AND (abstract: (Depression) OR abstract: ("Depressive Symptoms") OR abstract: ("Depressive Symptom") OR abstract: ("Emotional Depression") OR abstract: ("central depression") OR abstract: ("clinical depression") OR abstract: ("depressive disease") OR abstract: ("Depressive Disorder") OR abstract: ("depressive episode") OR abstract: ("depressive illness") OR abstract: ("depressive personality disorder") OR abstract: ("depressive state") OR abstract: ("Depressive Symptom") OR abstract: ("Depressive Syndrome") OR abstract: ("mental depression") OR abstract: ("parental depression") OR abstract: ("Major Depression") OR abstract: ("late life depression") OR abstract: ("late life depression") OR abstract: ("Depressive Disorder") OR abstract: ("Depressive Disorders") OR abstract: ("Depressive Neuroses") OR abstract: ("Depressive Neurosis") OR abstract: ("Endogenous Depression") OR abstract: ("Endogenous Depressions") OR abstract: ("Depressive Syndrome") OR abstract: ("Depressive Syndromes") OR abstract: ("Neurotic Depression") OR abstract: ("Neurotic Depressions") OR abstract: ("Melancholia") OR abstract: ("Melancholias") OR abstract: ("Unipolar Depression") OR abstract: ("Unipolar Depressions") OR abstract: ("Major Depressive Disorders") OR abstract: ("Major Depressive Disorder") OR abstract: ("Involutional Psychoses") OR abstract: ("Involutional Psychosis") OR abstract: ("Involutional Depression") OR abstract: ("Involutional Melancholia") OR abstract: (Anxiety) OR abstract: (Angst) OR abstract: ("Social Anxiety") OR abstract: ("Social Anxieties") OR abstract: (Hypervigilance) OR abstract: (Nervousness) OR abstract: (Anxiousness) OR abstract: (Apprehension) OR abstract: (Worry) OR abstract: ("Anxiety Disorders") OR abstract: ("Anxiety Disorder") OR abstract: ("Anxiety Neuroses") OR abstract: ("Neurotic Anxiety States") OR abstract: ("Anxiety Neurosis") OR abstract: ("Neurotic Anxiety State") OR abstract: ("Involutional Paraphrenia") OR abstract: ("Involutional Paraphrenias")) AND (abstract: (Aged) OR abstract: (Elderly) OR abstract: ("80 and over") OR abstract: ("Oldest Old") OR abstract: (Nonagenarian) OR abstract: (Nonagenarians) OR abstract: (Octogenarians) OR abstract: (Octogenarian) OR abstract: (Centenarians) OR abstract: (Centenarian) OR abstract: (geriatric) OR abstract: ("Middle Aged") OR abstract: ("Middle Age"))) OR ((title: (Dancing) OR title: (Dance) OR title: (danced) OR title: (dances) OR title: (dancers) OR title: (Ballet) OR title: ("Square Dance") OR title: ("hip hop") OR title: (Jazz) OR title: ("Tap Dance") OR title: ("Modern Dance") OR title: (Salsa) OR title: ("Line Dancing") OR title: ("social dance") OR title: (Tango) OR title: (ballroom) OR title: ("Irish set") OR title: ("Square Dance") OR title: (waltz) OR title: ("Aerobic dancing") OR title: ("Ballroom dancing") OR title: ("Belly dance") OR title: ("Break dancing") OR title: ("Cha-cha") OR title: (Flamenco) OR title: ("Folk dancing") OR title: ("Ice dancing") OR title: ("Pole dancing") OR title: ("Step dancing") OR title: (Rumba) OR title: (Blues) OR title: (Jitterbug) OR title: (Viennese) OR title: (Foxtrot) OR title: (Swing) OR title: (Merengue) OR title: (Disco) OR title: ("Dance Therapy") OR title: ("Dance Therapies") OR title: ("therapeutic dance") OR title: ("Movement Therapy") OR title: ("Dance movement therapy")) AND (title: (Depression) OR title: ("Depressive Symptoms") OR title: ("Depressive Symptom") OR title: ("Emotional Depression") OR title: ("central depression") OR title: ("clinical depression") OR title: ("depressive disease") OR title: ("Depressive Disorder") OR title: ("depressive episode") OR title: ("depressive illness") OR title: ("depressive personality disorder") OR title: ("depressive state") OR title: ("Depressive Symptom") OR title: ("Depressive Syndrome") OR title: ("mental depression") OR title: ("parental depression") OR title: ("Major Depression") OR title: ("late life depression") OR title: ("late life depression") OR title: ("Depressive Disorder") OR title: ("Depressive Disorders") OR title: ("Depressive Neuroses") OR title: ("Depressive Neurosis") OR title: ("Endogenous Depression") OR title: ("Endogenous Depressions") OR title: ("Depressive Syndrome") OR title: ("Depressive Syndromes") OR title: ("Neurotic Depression") OR title: ("Neurotic Depressions") OR title: ("Melancholia") OR title: ("Melancholias") OR title: ("Unipolar Depression") OR title: ("Unipolar Depressions") OR title: ("Major Depressive Disorders") OR title: ("Major Depressive Disorder") OR title: ("Involutional Psychoses") OR title: ("Involutional Psychosis") OR title: ("Involutional Depression") OR title: ("Involutional Melancholia") OR title: (Anxiety) OR title: (Angst) OR title: ("Social Anxiety") OR title: ("Social Anxieties") OR title: (Hypervigilance) OR title: (Nervousness) OR title: (Anxiousness) OR title: (Apprehension) OR title: (Worry) OR title: ("Anxiety Disorders") OR title: ("Anxiety Disorder") OR title: ("Anxiety Neuroses") OR title: ("Neurotic Anxiety States") OR title: ("Anxiety Neurosis") OR title: ("Neurotic Anxiety State") OR title: ("Involutional Paraphrenia") OR title: ("Involutional Paraphrenias")) AND (title: (Aged) OR title: (Elderly) OR title: ("80 and over") OR title: ("Oldest Old") OR title: (Nonagenarian) OR title: (Nonagenarians) OR title: (Octogenarians) OR title: (Octogenarian) OR title: (Centenarians) OR title: (Centenarian) OR title: (geriatric) OR title: ("Middle Aged") OR title: ("Middle Age")))	
**SPORTDiscus (EBSCO)**	(Dancing OR Dance OR danced OR dances OR dancers OR Ballet OR "Square Dance" OR "hip hop" OR Jazz OR "Tap Dance" OR "Modern Dance" OR Salsa OR "Line Dancing" OR "social dance" OR Tango OR ballroom OR "Irish set" OR "Square Dance" OR waltz OR "Aerobic dancing" OR	35

	"Ballroom dancing" OR "Belly dance" OR "Break dancing" OR "Cha-cha" OR Flamenco OR "Folk dancing" OR "Ice dancing" OR "Pole dancing" OR "Step dancing" OR Rumba OR Blues OR Jitterbug OR Viennese OR Foxtrot OR Swing OR Merengue OR Disco OR "Dance Therapy" OR "Dance Therapies" OR "therapeutic dance" OR "Movement Therapy" OR "Dance movement therapy") AND (Depression OR "Depressive Symptoms" OR "Depressive Symptom" OR "Emotional Depression" OR "central depression" OR "clinical depression" OR "depressive disease" OR "Depressive Disorder" OR "depressive episode" OR "depressive illness" OR "depressive personality disorder" OR "depressive state" OR "Depressive Symptom" OR "Depressive Syndrome" OR "mental depression" OR "parental depression" OR "Major Depression" OR "late life depression" OR "late life depression" OR "Depressive Disorder" OR "Depressive Disorders" OR "Depressive Neuroses" OR "Depressive Neurosis" OR "Endogenous Depression" OR "Endogenous Depressions" OR "Depressive Syndrome" OR "Depressive Syndromes" OR "Neurotic Depression" OR "Neurotic Depressions" OR "Melancholia" OR "Melancholias" OR "Unipolar Depression" OR "Unipolar Depressions" OR "Major Depressive Disorders" OR "Major Depressive Disorder" OR "Involutional Psychoses" OR "Involutional Psychosis" OR "Involutional Depression" OR "Involutional Melancholia" OR Anxiety OR Angst OR "Social Anxiety" OR "Social Anxieties" OR Hypervigilance OR Nervousness OR Anxiousness OR Apprehension OR Worry OR "Anxiety Disorders" OR "Anxiety Disorder" OR "Anxiety Neuroses" OR "Neurotic Anxiety States" OR "Anxiety Neurosis" OR "Neurotic Anxiety State" OR "Involutional Paraphrenia" OR "Involutional Paraphrenias") AND (Aged OR Elderly OR "80 and over" OR "Oldest Old" OR Nonagenarian OR Nonagenarians OR Octogenarians OR Octogenarian OR Centenarians OR Centenarian OR geriatric OR "Middle Aged" OR "Middle Age")	
**LILACS**	(dancing OR dance OR danced OR dances OR dancers OR ballet OR "Square Dance" OR "hip hop" OR jazz OR "Tap Dance" OR "Modern Dance" OR salsa OR "Line Dancing" OR "social dance" OR tango OR ballroom OR "Irish set" OR "Square Dance" OR waltz OR "Aerobic dancing" OR "Ballroom dancing" OR "Belly dance" OR "Break dancing" OR "Cha-cha" OR flamenco OR "Folk dancing" OR "Ice dancing" OR "Pole dancing" OR "Step dancing" OR rumba OR blues OR jitterbug OR viennese OR foxtrot OR swing OR merengue OR disco OR "Dance Therapy" OR "Dance Therapies" OR "therapeutic dance" OR "Movement Therapy" OR "Dance movement therapy" OR dança OR balé OR "Dança Moderna" OR "Dança Salsa" OR quadrilha OR "Dança em Linha" OR sapateado OR "Square Dance" OR baile OR bailes OR cuadrilla OR danza OR danzas OR zapateado OR dançaterapia OR "Psicoterapia através da Dança" OR "Terapia através da Dança" OR "Terapia pela Dança" OR danzaterapia OR danzoterapia OR "Terapia a través de la Danza" OR "terapia por la danza" OR "tratamiento por la danza") AND (depression OR "Depressive Symptoms" OR "Depressive Symptom" OR "Emotional Depression" OR "central depression" OR "clinical depression" OR "depressive disease" OR "Depressive Disorder" OR "depressive episode" OR "depressive illness" OR "depressive personality disorder" OR "depressive state" OR "Depressive Symptom" OR "Depressive Syndrome" OR "mental depression" OR "parental depression" OR "Major Depression" OR "late life depression" OR "late life depression" OR "Depressive Disorder" OR "Depressive Disorders" OR "Depressive Neuroses" OR "Depressive Neurosis" OR "Endogenous Depression" OR "Endogenous Depressions" OR "Depressive Syndrome" OR "Depressive Syndromes" OR "Neurotic Depression" OR "Neurotic Depressions" OR "Melancholia" OR "Melancholias" OR "Unipolar Depression" OR "Unipolar Depressions" OR "Major Depressive Disorders" OR "Major Depressive Disorder" OR "Involutional Psychoses" OR "Involutional Psychosis" OR "Involutional Depression" OR "Involutional Melancholia" OR anxiety OR angst OR "Social Anxiety" OR "Social Anxieties" OR hypervigilance OR nervousness OR anxiousness OR apprehension OR worry OR "Anxiety Disorders" OR "Anxiety Disorder" OR "Anxiety Neuroses" OR "Neurotic Anxiety States" OR "Anxiety Neurosis" OR "Neurotic Anxiety State" OR "Involutional Paraphrenia" OR "Involutional Paraphrenias" OR depressão OR "Sintomas Depressivos" OR depresión OR "Síntomas Depresivos" OR "Transtorno Depressivo" OR "Depressão Endógena" OR "Depressão Neurótica" OR "Depressão Unipolar" OR melancolia OR "Neurose Depressiva" OR "Síndrome Depressiva" OR "Transtornos Depressivos" OR "Trastorno Depresivo" OR "Depresión Endógena" OR "Depresión Neurótica" OR "Depresión Unipolar" OR "Neurosis Depresiva" OR "Síndrome Depresivo" OR "Transtorno Depressivo Maior" OR "Depressão Involutiva" OR "Melancolia Involutiva" OR "Parafrenia Involutiva" OR "Psicose Involutiva" OR "Trastorno Depresivo Mayor" OR "Depresión Involutiva" OR "Melancolía Involutiva" OR "Parafrenia Involutiva" OR "Psicosis Involutiva" OR "trastorno de depresión mayor" OR ansiedade OR angustia OR hipervigilancia OR nervosismo OR ansiedad OR nerviosismo OR "Transtornos de Ansiedade" OR "Distúrbios de Ansiedade" OR "Estados de Ansiedade Neurótica" OR "Neurose de Ansiedade" OR "Transtornos Ansiosos" OR "Transtornos de Angústia" OR "Trastornos de Ansiedad" OR "Estados de Ansiedad Neurótica" OR "Trastornos de Angustia" OR "Neurosis de Ansiedad") AND (aged OR elderly OR "80 and over" OR "Oldest	31

	Old" OR nonagenarian OR nonagenarians OR octogenarians OR octogenarian OR centenarians OR centenarian OR geriatric OR "Middle Aged" OR "Middle Age" OR idoso OR idosos OR idosa OR idosas OR "Pessoa de Idade" OR "Pessoas de Idade" OR anciano OR ancianos OR "Adulto Mayor" OR "Persona Mayor" OR "Persona de Edad" OR "Personas Mayores" OR "Personas de Edad" OR "Idoso de 80 Anos ou mais" OR centenarios OR nonagenarios OR octogenarios OR velhíssimos OR "Anciano de 80 o más Años" OR viejísimos OR geriátrico OR geriátricos OR geriátrica OR geriátricas OR "Meia Idade" OR "Mediana Edad") AND (db:("LILACS"))	
**ProQuest Dissertations & Theses Global**	noft(Dancing OR Dance OR danced OR dances OR dancers OR Ballet OR "Square Dance" OR "hip hop" OR Jazz OR "Tap Dance" OR "Modern Dance" OR Salsa OR "Line Dancing" OR "social dance" OR Tango OR ballroom OR "Irish set" OR "Square Dance" OR waltz OR "Aerobic dancing" OR "Ballroom dancing" OR "Belly dance" OR "Break dancing" OR "Cha-cha" OR Flamenco OR "Folk dancing" OR "Ice dancing" OR "Pole dancing" OR "Step dancing" OR Rumba OR Blues OR Jitterbug OR Viennese OR Foxtrot OR Swing OR Merengue OR Disco OR "Dance Therapy" OR "Dance Therapies" OR "therapeutic dance" OR "Movement Therapy" OR "Dance movement therapy") AND noft(Depression OR "Depressive Symptoms" OR "Depressive Symptom" OR "Emotional Depression" OR "central depression" OR "clinical depression" OR "depressive disease" OR "Depressive Disorder" OR "depressive episode" OR "depressive illness" OR "depressive personality disorder" OR "depressive state" OR "Depressive Symptom" OR "Depressive Syndrome" OR "mental depression" OR "parental depression" OR "Major Depression" OR "late life depression" OR "late life depression" OR "Depressive Disorder" OR "Depressive Disorders" OR "Depressive Neuroses" OR "Depressive Neurosis" OR "Endogenous Depression" OR "Endogenous Depressions" OR "Depressive Syndrome" OR "Depressive Syndromes" OR "Neurotic Depression" OR "Neurotic Depressions" OR "Melancholia" OR "Melancholias" OR "Unipolar Depression" OR "Unipolar Depressions" OR "Major Depressive Disorders" OR "Major Depressive Disorder" OR "Involutional Psychoses" OR "Involutional Psychosis" OR "Involutional Depression" OR "Involutional Melancholia" OR Anxiety OR Angst OR "Social Anxiety" OR "Social Anxieties" OR Hypervigilance OR Nervousness OR Anxiousness OR Apprehension OR Worry OR "Anxiety Disorders" OR "Anxiety Disorder" OR "Anxiety Neuroses" OR "Neurotic Anxiety States" OR "Anxiety Neurosis" OR "Neurotic Anxiety State" OR "Involutional Paraphrenia" OR "Involutional Paraphrenias") AND noft(Aged OR Elderly OR "80 and over" OR "Oldest Old" OR Nonagenarian OR Nonagenarians OR Octogenarians OR Octogenarian OR Centenarians OR Centenarian OR geriatric OR "Middle Aged" OR "Middle Age")	49
**LIVIVO**	(Dancing OR Dance OR danced OR dances OR dancers OR Ballet OR "Square Dance" OR "hip hop" OR Jazz OR "Tap Dance" OR "Modern Dance" OR Salsa OR "Line Dancing" OR "social dance" OR Tango OR ballroom OR "Irish set" OR "Square Dance" OR waltz OR "Aerobic dancing" OR "Ballroom dancing" OR "Belly dance" OR "Break dancing" OR "Cha-cha" OR Flamenco OR "Folk dancing" OR "Ice dancing" OR "Pole dancing" OR "Step dancing" OR Rumba OR Blues OR Jitterbug OR Viennese OR Foxtrot OR Swing OR Merengue OR Disco OR "Dance Therapy" OR "Dance Therapies" OR "therapeutic dance" OR "Movement Therapy" OR "Dance movement therapy") AND (Depression OR "Depressive Symptoms" OR "Depressive Symptom" OR "Emotional Depression" OR "central depression" OR "clinical depression" OR "depressive disease" OR "Depressive Disorder" OR "depressive episode" OR "depressive illness" OR "depressive personality disorder" OR "depressive state" OR "Depressive Symptom" OR "Depressive Syndrome" OR "mental depression" OR "parental depression" OR "Major Depression" OR "late life depression" OR "late life depression" OR "Depressive Disorder" OR "Depressive Disorders" OR "Depressive Neuroses" OR "Depressive Neurosis" OR "Endogenous Depression" OR "Endogenous Depressions" OR "Depressive Syndrome" OR "Depressive Syndromes" OR "Neurotic Depression" OR "Neurotic Depressions" OR "Melancholia" OR "Melancholias" OR "Unipolar Depression" OR "Unipolar Depressions" OR "Major Depressive Disorders" OR "Major Depressive Disorder" OR "Involutional Psychoses" OR "Involutional Psychosis" OR "Involutional Depression" OR "Involutional Melancholia" OR Anxiety OR Angst OR "Social Anxiety" OR "Social Anxieties" OR Hypervigilance OR Nervousness OR Anxiousness OR Apprehension OR Worry OR "Anxiety Disorders" OR "Anxiety Disorder" OR "Anxiety Neuroses" OR "Neurotic Anxiety States" OR "Anxiety Neurosis" OR "Neurotic Anxiety State" OR "Involutional Paraphrenia" OR "Involutional Paraphrenias") AND (Aged OR Elderly OR "80 and over" OR "Oldest Old" OR Nonagenarian OR Nonagenarians OR Octogenarians OR Octogenarian OR Centenarians OR Centenarian OR geriatric OR "Middle Aged" OR "Middle Age")	1,523
**Google Scholar**	(Dancing OR Dance OR "Movement Therapy") AND (Depression OR Depressive OR Anxiety) AND (Aged OR Elderly OR geriatric)	100

Search strategies were performed for each database by using specifics words combinations and truncations with the support of a librarian.

Table S2. Certainty of evidence

Author(s): Tiago Paiva Prudente, Eleazar Mezaiko, Erika Aparecida da Silveira, Túlio Eduardo Nogueira
Question: What is the effect of dance on symptoms of depression and anxiety in older adults?

Certainty assessment							№ of patients		Effect		Certainty	Importance
№ of studies	Study design	Risk of bias	Inconsistency	Indirectness	Imprecision	Other considerations	dance	no dance	Relative (95% CI)	Absolute (95% CI)		
**Anxiety**												
4	randomized trials	serious	serious	serious	not serious	publication bias strongly suspected	150	109	-	SMD 1.81 SD lower (3.59 lower to 0.04 lower)	⊕◯◯◯ Very low	IMPORTANT
**Depression - Dance vs No intervention**												
8	randomized trials	not serious	serious	serious	serious	none	256	251	-	SMD 0.78 SD lower (1.45 lower to 0.11 lower)	⊕◯◯◯ Very low	IMPORTANT
**Depression - Dance vs Other interventions**												
6	randomized trials	serious	serious	serious	serious	none	94	101	-	SMD 0.01 SD higher (0.47 lower to 0.49 higher)	⊕◯◯◯ Very low	IMPORTANT

CI: confidence interval; **SMD:** standardized mean difference
